# Survey of familial glaucoma shows a high incidence of cytochrome P450, family 1, subfamily B, polypeptide 1 (*CYP1B1*) mutations in non-consanguineous congenital forms in a Spanish population

**Published:** 2013-08-04

**Authors:** Elena Millá, Begoña Mañé, Susana Duch, Imma Hernan, Emma Borràs, Ester Planas, Miguel de Sousa Dias, Miguel Carballo, María José Gamundi

**Affiliations:** 1 Molecular Genetics Unit, Hospital of Terrassa (Spain), Ctra. Torrebonica s/n, 08227 Terrassa, Barcelona, Spain; 2 Unidad de Glaucoma y Genética, Institut Comtal d’Oftalmologia, Barcelona, Spain; 3 Unidad de Glaucoma, Institut Clínic d’Oftalmologia (ICOF), Hospital Clínic, Barcelona, Spain; 4 Unidad de Glaucoma, Institut Comtal d'Oftalmologia (ICO), Barcelona, Spain.

## Abstract

**Purpose:**

To identify myocilin (*MYOC*) and cytochrome P450, family 1, subfamily B, polypeptide 1 (*CYP1B1*) mutations in a Spanish population with different clinical forms of familial glaucoma or ocular hypertension (OHT).

**Methods:**

Index patients from 226 families participated in this study. Patients were diagnosed with familial glaucoma or OHT by complete ophthalmologic examination. Screening for *MYOC* mutations was performed in 207 index patients: 96 with adult-onset primary open-angle glaucoma (POAG), 21 with primary congenital glaucoma (PCG), 18 with juvenile-onset open-angle glaucoma (JOAG), five with Axenfeld-Rieger syndrome (ARS), and 67 with other types of glaucoma. One hundred two of the families (including all those in whom a *MYOC* mutation was detected) were also screened for *CYP1B1* mutations: 45 POAG, 25 PCG, 21 JOAG, four ARS, and seven others.

**Results:**

We examined 292 individuals (patients and relatives) with a positive family history of glaucoma or OHT. We identified two novel *MYOC* variants, p.Lys39Arg and p.Glu218Lys, in two families with POAG, and six previously reported *MYOC* mutations in seven families with POAG (four), JOAG (one), PCG (one), and normotensive glaucoma (one). *CYP1B1* mutations were found in 16 index patients with PCG (nine), POAG (three), JOAG (two), and ARS (two).

**Conclusions:**

The high percentage (9/25=36%) of mutations in *CYP1B1* found in non-consanguineous patients with congenital glaucoma mandates genetic testing. However, the percentage of mutations (9/207=4.4%) in *MYOC* associated with glaucoma is relatively low in our population. The variable phenotype expression of glaucoma, even in families, cannot be explained with a digenic mechanism between *MYOC* and *CYP1B1*.

## Introduction

Glaucoma comprises a heterogeneous group of progressive optic neuropathies characterized by excavation of the optic disc and progressive alteration of the visual field [1]. High intraocular pressure (IOP) and a positive family history for glaucoma are commonly associated risk factors. Based on the age of onset and other clinical features, glaucoma has been classified as primary congenital glaucoma (PCG), juvenile-onset open-angle glaucoma (JOAG), and adult-onset primary open-angle glaucoma (POAG). As Lander and Schork remarked [2], glaucoma is a complex trait, in most cases does not follow a clear-cut inheritance pattern, and has variable penetrance, insidious progression, and usually a late onset. Linkage analyses have identified 23 loci (GLC1A-GLC1L, GLC3A-GLC3B, 2p14, 2q33-q34, 5q22.1-q32, 10p12-p13, 14q11, 14q21-q22, 17p13, 17q25, and 19q12-q14) [3-8] for different forms of glaucoma. However, only four genes (*MYOC/TIGR*, *CYP1B1*, optineurin [*OPTN*], and WD repeat domain 36 [*WDR36*] [6]; ) have been identified thus far.

Mutations in the *MYOC* gene have been associated with POAG and JOAG [9]. More than 90 point mutations have been identified worldwide in multiple ethnic groups [10]. These mutations account for 3% to 5% of POAG cases and a larger proportion of JOAG cases (approximately 6% to 36%) [7,8]. The *MYOC* gene contains three exons and codes for a 504-aa protein [11]. Most mutations are in the third exon of *MYOC* [9,11]. The function of myocilin is unknown, but it is expressed ubiquitously in the eye.

Primary congenital glaucoma is a rare form of glaucoma and is usually transmitted as an autosomal recessive disease with incomplete penetrance [12]. PCG is the most common childhood glaucoma, and one of the most important causes of blindness in children. Three PCG loci have been mapped, but cytochrome P450, family 1, subfamily B, polypeptide 1 (*CYP1B1*) is the only gene identified to date. More than 150 mutations including missense, nonsense, regulatory, and insertions and/or deletions in *CYP1B1* have been associated with PCG [10], and are the main known cause of PCG. The *CYP1B1* gene contains three exons, and the coding region begins at the second exon. The 543-aa *CYP1B1* protein belongs to the cytochrome P450 family, a group of heme-thiolate monooxygenases. In liver microsomes, this enzyme is involved in a nicotinamide adenine dinucleotide phosphate (NADP)-oxidase–dependent electron transport pathway. The enzyme oxidizes various structurally unrelated compounds, including steroids, fatty acids, and xenobiotics. *CYP1B1* participates in the metabolism of an as-yet-unknown biologically active molecule that participates in eye development [13]. Another gene possibly associated with PCG is latent transforming growth factor beta binding protein 2 (*LTBP2*)*.* This gene maps to chromosome 14q24.3 but is around 1.3 Mb proximal to the documented glaucoma 3, primary congenital, C (*GLC3C*) locus. Therefore, whether *LTBP2* is the *GLC3C* gene or whether a second adjacent gene is also implicated in PCG remains to be determined [11]. Mutations in *LTBP2* have been reported to cause PCG in some populations [14,15].

Glaucoma is a treatable disease if detected early [16]. Consequently, development of an accurate test for detecting presymptomatic carriers at risk is important for managing glaucoma. Previous studies have shown that 34% of glaucoma suspect patients had a positive family history. The actual percentage of patients with POAG with affected relatives could be higher because 50% of individuals with POAG are unaware of their clinical status, and a much higher percentage (56%) of probands with POAG have affected relatives whose disease status is determined by clinical examination rather than by family history alone [17]. Family history is important clinically because the risk for POAG among first-degree relatives of a patient with POAG is 7–10 times higher than that of the general population, and surveillance targeting of these individuals is indicated for early detection and treatment of POAG [18].

Based upon these considerations, we started a study for identifying myocilin (*MYOC)* and cytochrome P450, family 1, subfamily B, polypeptide 1 (*CYP1B1*) mutations in a Spanish population with different clinical forms of familial glaucoma or ocular hypertension (OHT). The objectives of this study were the clinical characterization of our population of patients with glaucoma or OHT, followed by the clinical characterization of relatives of patients with glaucoma or OHT to detect undiagnosed cases. We then performed a mutational analysis of *MYOC* and *CYP1B1* in patients and their relatives to establish a genotype-phenotype correlation and detect presymptomatic carriers at risk to establish a better therapeutic approach for these cases. With these results, the aim was to modulate glaucoma treatment according to the genetic result and provide genetic counseling.

## Methods

Before being included in this study, all the participants were informed of its objectives. All gave their informed consent to participate in the study, which adhered to the tenets of the Declaration of Helsinki. All were asked to complete a questionnaire that included personal, biographic, demographic, family, and clinical data.

The patients were enrolled by their treating ophthalmologists from 18 different Spanish hospitals in a collaborative study named Estudio Multicéntrico Español de Investigación Genética del Glaucoma (EMEIGG). All the patients included in the study had been diagnosed with glaucoma or OHT after a complete ophthalmologic study that included best corrected visual acuity (BCVA), tested with Snellen charts, and ocular refraction, evaluated with an autorefractometer. Biomicroscopy of the anterior segment was performed. Tonometry was performed with a Goldmann applanation tonometer, using the mean of three consecutive tonometry readings for each eye. Gonioscopy with a four-mirror lens and fundoscopy for the observation of the optic nerve head and peripapillary region under direct examination with a 78 D Volkmann lens completed the slit-lamp exam. As controls, 119 samples were taken from subjects with no family history of glaucoma and aged >60 years, recruited at routine ophthalmic examinations, and screened for mutations.

### Ancillary examinations

All cases underwent computerized perimetry with a 750-Humphrey field analyzer II following a SITA Standard strategy, size III stimulus and a white-on-white test. Monoscopic ONH and peripapillary RNFL digital pictures were taken with a Topcon nonmydriatic retinal camera (Topcon model TRC-NW6S, Topcon, Tokyo, Japan). Ultrasound pachymetry was performed under topical anesthesia with an Ocuscan RxP pachymeter (Alcon Laboratories, Irvine, CA) that provides six central readings and calculates the mean value. The IOP is then manually introduced in each case, and the machine automatically provides a new IOP value corrected for the pachymetry measurements (Herndon formula). Optical coherence tomography was performed with OCT version 3 (Stratus OCT, Carl Zeiss Meditec, Dublin, CA) in the glaucoma suspects and Heidelberg retina tomograph (HRT-II) in the glaucoma subjects. All individuals received pupillary dilatation with 0.5% tropicamide.

### Mutation analysis of coding exons of myocilin and cytochrome P450, family 1, subfamily B, polypeptide 1

Genomic DNA was isolated from peripheral blood samples of the patients with the MagNA Pure Compact Nucleic Acid Isolation Kit I using the MagNA Pure Compact System (Roche, Barcelona, Spain) according to the manufacturer’s protocol. Coding exons of *MYOC* and *CYP1B1* were amplified with PCR using the primers and conditions listed in Table 1. PCR reactions were performed in a total volume of 50 μl using 1X NH_4_ buffer, 1.5 mM MgCl_2_, and 10% dimethyl sulfoxide, 0.2 mM deoxyribonucleoside triphosphate, 2.5 U of BIOTAQ DNA Polymerase (Ecogen, Barcelona, Spain), 0.5 μM of each primer, and 200–500 ng of human genomic DNA. Thermal cycling consisted of denaturation at 95 °C for 5 min, followed by 35 cycles of denaturation at 95 °C for 30 s, annealing for 30 s and extension at 72 °C for 1 min. PCR fragments were purified with High Pure PCR Product Purification Kit (Roche) and sequenced by Stab Vida (Oeiras, Portugal) on a 3730XL ABI DNA sequencer (Applied Biosystems) using the Big Dye terminator V1.1 DNA sequencing kit. Chromatograms were analyzed and compared with RefSeq sequences (*MYOC:* ENSG00000034971, *CYP1B1:* ENSG00000138061) to detect any variation in the sequence. To detect a possible deletion in chromosome 2, 23 chromosome markers were analyzed in all the members of family PCG-47 (Appendix 1).

**Table 1:** Primers and PCR conditions used for the amplification of coding exons of *MYOC* and *CYP1B1.*

Fragment	Primer	Sequence (5’-3’)	Annealing temperature	Amplicon size (bp)
*MYOC-1*	MYOC-1F	CTCTGGAGCTCGGGCATG	56.9 °C	753
	MYOC-1R	TCACTACGAGCCATATCACC		
*MYOC-2*	MYOC-2F	CATAGTCAATCCTTGGGC	54.5 °C	380
	MYOC-2R	CTGCAGACCTGCTCTGACAA		
*MYOC-3A* *MYOC-3B*	MYOC-3AF	GATTTGTCTCCAGGGCTGTC	58.4 °C	724
	MYOC-3AR	CTGCTGAGGTGTAGCTGCTG		
	MYOC-3BF	TCTGTGGCACCTTGTACACC	56 °C	994
	MYOC-3BR	ACATCTCCCAACCTGAAGGAA		
*CYP1B1–2*	CYP1B1–3F	GGCGCCCGCTCCTGTCTCTG	61.4 °C	1216
	CYP1B1–3R	ACCTGGAGCGAAACCCCAAA		
*CYP1B1–3*	CYP1B1–4F	AATGTGCTTTCTAGATGAAATAAGAA	54.6 °C	751
	CYP1B1–4R	CAGCACAAAAGAGGAACTGGA		

## Results

### Analysis of myocilin mutations in patients with glaucoma

The three coding exons of *MYOC* were analyzed with PCR and sequencing in 207 index patients with various types of glaucoma (Table 2) and in 119 control individuals with no family history of ocular disorders. Eight mutations were identified in nine unrelated index patients with glaucoma, and the mutation analysis was extended to their families. In addition to these mutations, nine previously reported polymorphisms were detected in patients and control individuals (Appendix 1). Genetic screening of the control individuals revealed no pathogenic mutation in *MYOC*.

**Table 2:** Number of index patients analyzed and distribution of mutations in *MYOC* and *CYP1B1* found in the families studied.

**Type of glaucoma**	***MYOC* analysis (n)**	**No. families with *MYOC* mutation**	***CYP1B1* analysis (n)**	**No. families with *CYP1B1* mutation**
Axenfeld-Rieger Syndrome	5	-	4	2/4 (50%)
Closed-angle glaucoma	7	-	-	-
Glaucoma suspect	1	-	-	-
Juvenile-onset open angle glaucoma	18	1/18 (5.6%)	21	2/21 (9.5%)
Normal tension glaucoma	33	1/33 (3%)	1	-
Ocular hypertension	16	1/16 (6.2%)	2	-
Pigmentary glaucoma	1	-	-	-
Primary congenital glaucoma	21	1/21 (4.8%)	25	9/25 (36%)
Primary open angle glaucoma	96	5/96 (5.2%)	45	3/45 (6.7%)
Pseudoexfoliative glaucoma	3	-	-	-
Unclassified	6	-	6	-
**Total index patients**	**207**	**9/207 (4.4%)**	**102**	**16/102 (15.7%)**

**Figure 1:**
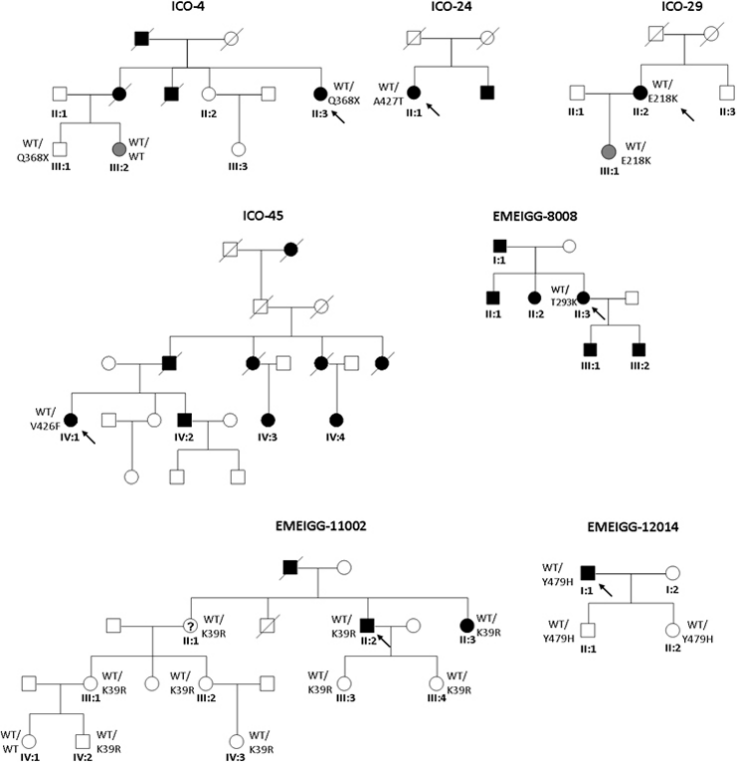
Pedigrees of glaucoma families carrying myocilin (*MYOC*) mutations. Filled symbols represent affected subjects, and unfilled symbols unaffected subjects. Squares indicate men, and circles women. Arrows reflect the index patients. WT depicts the wild-type allele.

In two index patients with POAG, we found two novel variants (p.Lys39Arg and p.Glu218Lys), and six previously reported [19-24] mutations in seven additional families, namely, p.Thr293Lys, p.Glu352Lys, p.Gln368Stop (in two families), p.Val426Phe, p.Ala427Thr, and p.Tyr479His (Table 3 and Figure 1).

**Table 3:** *MYOC* mutations detected in the families studied.

Family No.	Subject No.	Phenotype	*MYOC* mutation	Treatment	Prognosis
ICO-4	II:3*	Severe POAG	p.Gln368Stop (HT)	*Aggressive surgery More frequent examinations Withdrawn	Bad visual prognosis
	III:1	Normal	p.Gln368Stop (HT)		
	III:2	OHT	-		
ICO-24	II:1*	NTG	p.Ala427Thr (HT)	*Filtering surgery	Bad
ICO-29	II:2*	Mild POAG	p.Glu218Lys (HT)	*Medical treatment	Good
	III:1	OHT	p.Glu218Lys (HT)	None	Good
ICO-45	IV:1*	JOAG	p.Val426Phe (HT)	*Filtering surgery	Bad
EMEIGG-8008	II:3*	POAG	p.Thr293Lys (HT)	*Medical treatment	Good
EMEIGG-11002	II:1	POAG?	p.Lys39Arg (HT)	*Laser trabeculoplasty	Bad
	II:2*	Unilateral POAG	p.Lys39Arg (HT)		
	II:3	POAG	p.Lys39Arg (HT)		
	III:1	Normal	p.Lys39Arg (HT)		
	III:2	Normal	p.Lys39Arg (HT)		
	III:3	Normal	p.Lys39Arg (HT)		
	III:4	Normal	p.Lys39Arg (HT)		
	IV:1	Normal	-		
	IV:2	Normal	p.Lys39Arg (HT)		
	IV:3	Normal	p.Lys39Arg (HT)		
EMEIGG-12014	I:1	POAG	p.Tyr479His (HT)	*Filtering surgery	Moderately impaired
	II:1	Normal	p.Tyr479His (HT)		
	II:2	Normal	p.Tyr479His (HT)		
EMEIGG-21007	21,007.1*	Severe PCG	p.Glu352Lys (HT)	*Tube implant	Bad
EMEIGG-23001	23,001.1*	POAG	p.Gln368Stop (HT)	*Medical treatment	Good but early onset

* indicates index patient; 21,007 and 23,001: trees not shown

We found two novel variants in our survey. The p.Glu218Lys mutation was found in a patient, II:2 (Figure 1, ICO-29), with mild POAG controlled with a single drug. His daughter (III:1) also carried the mutation and had OHT. Her eye examinations were all unremarkable except a borderline OCT reading in the superior peripapillary area. The second novel variant found in MYOC, c.116A>G, which creates the p.Lys39Arg substitution, was found in index patient II:2 (family EMEIGG-11002, Figure 1). This patient presented with late-onset unilateral POAG that was controlled with argon laser trabeculoplasty and medications; the other eye was unremarkable. All this patient’s relatives except one (see Figure 1 and Table 3) are heterozygous carriers of the variant. We evaluated the pathogenesis of the novel *MYOC* variants detected, p.Lys39Arg and p.Glu218Lys, using the prediction algorithm the Sorting Intolerant From Tolerant (SIFT) and polymorphism phenotyping (PolyPhen) (Appendix 1) and found only as probably damaging p.Glu218Lys. Both variants were absent in the 119 controls and not yet reported in the 1000 Genomes database.

### Analysis of cytochrome P450, family 1, subfamily B, polypeptide 1 mutations in patients with glaucoma

Mutations in *CYP1B1* have been reported to cause glaucoma in a recessive trait. The two coding exons of *CYP1B1* were analyzed with PCR and sequencing in 45 index patients with POAG, 25 index patients with PCG, 21 index patients with JOAG, four index patients with Axenfeld-Rieger syndrome (ARS), and seven index patients with other types of glaucoma (Table 2). *CYP1B1* mutation screening was performed in 119 control individuals with no family history of glaucoma. All these patients were previously screened for mutations in *MYOC*.

**Figure 2:**
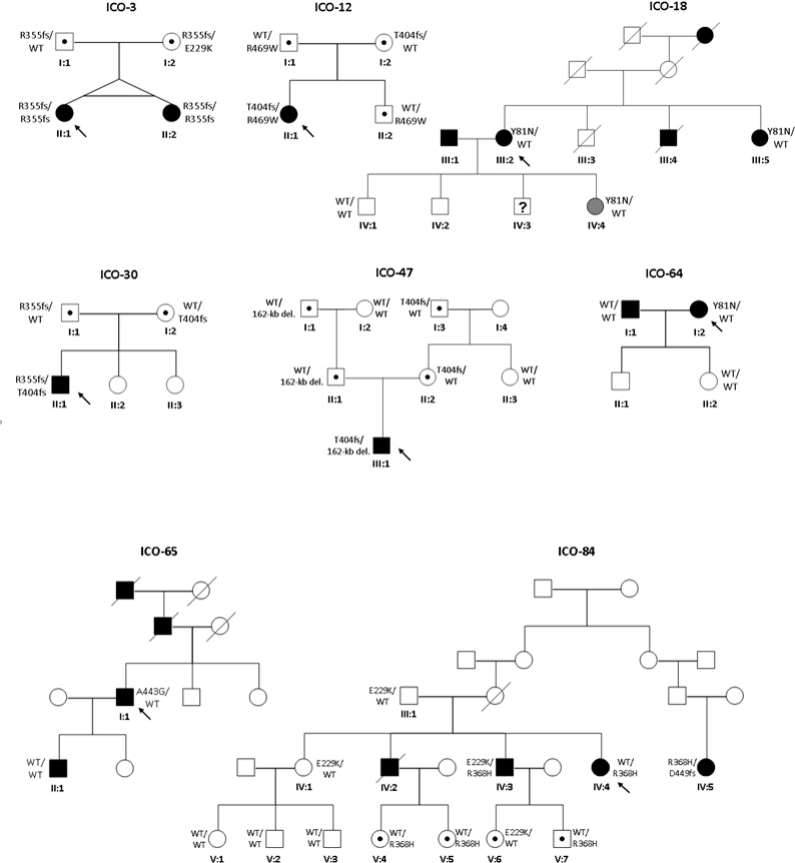
Pedigrees of glaucoma families from the Institut Comtal d’Oftalmologia (ICO) carrying mutations in *CYP1B1*. Filled symbols represent affected subjects, and unfilled symbols unaffected subjects. Squares indicate men, and circles women. Arrows represent the index patients. WT depicts the wild-type allele.

Twelve previously reported *CYP1B1* mutations [25-35] and a novel variant (c.1403_1429dup, p.Arg468_Ser476dup; Table 4 and Table 5) were initially detected in nine index patients with PCG, three with POAG, two with JOAG and two with ARS (Table 4 and Table 5). When we analyzed relatives of the index patients (Figure 2), an additional variant (c. 1344_1345delG, p.Asp449fsX6) was detected in a family with PCG (family ICO-84). Most of the patients with PCG were carriers of homozygous or compound heterozygous mutations in *CYP1B1*; however, in three patients with PCG, only one mutation was detected (Table 4 and Table 5). In addition to these mutations, eight previously reported polymorphisms were detected in patients and the control individuals (Appendix 1). In the control population assayed (n=119), three previously reported mutations (p.Trp57Stop, p.Glu229Lys, and p.Trp341Stop reported as a null allele) [35] were detected in heterozygosis in three independent individuals.

**Table 4:** *CYP1B1* mutations detected in the PCG and ARS families studied from ICO.

Family No.	Patient No.	Phenotype	Mutation 1	Mutation 2	Treatment and prognosis
ICO-3	I:1	Normal	p.Arg355fsX69	-	*Molteno tube implantation and cyclodestruction and severe visual impairment in one eye and good response to goniotomies and medical treatment in the other: both twins
	I:2	Normal	p.Arg355fsX69	p.Glu229Lys	
	II:1*	Severe PCG	p.Arg355fsX69	p.Arg355fsX69	
	II:2	Severe PCG	p.Arg355fsX69	p.Arg355fsX69	
ICO-12	I:1	Normal	-	p.Arg469Trp	*Molteno tube implantation in both eyes during childhood
	I:2	Normal	p.Thr404fsX30	-	
	II:1*	Severe PCG	p.Thr404fsX30	p.Arg469Trp	
	II:2	Normal	-	p.Arg469Trp	
ICO-18	III:2*	POAG	p.Tyr81Asn	-	*Severe Pseudoexfoliative glaucoma requiring filtration surgery in both eyes
	III:5	PEX	p.Tyr81Asn	-	
	IV:1	OHT	-	-	
	IV:4	Normal	p.Tyr81Asn	-	
ICO-19	G-41*	Severe unilateral PCG	p.Gly61Glu	Not detected	Conventional filtering surgery/ Vision badly impaired to finger counting in RE/ good response to goniotomy with normal VA LE
ICO-30	I:1	Normal	p.Arg355fsX69	-	Bilateral tube implantation, severe phenotype in both eyes
	I:2	Normal	-	p.Thr404fsX30	
	II:1	Severe bilateral PCG	p.Arg355fsX69	p.Thr404fsX30	
ICO-39	G-74*	JOAG	p.Arg368His	p.Thr404fsX30	Severe phenotype that required filtering surgery (trabeculectomy) in both eyes
ICO-47	I:1	Normal	-	162-kb del(2p21.1)	*Goniotomy and tube implantation bilaterally, severe phenotype
	I:2	Normal	-	-	
	I:3	Normal	p.Thr404fsX30	-	
	II:1	Normal	-	162-kb del(2p21.1)	
	II:2	Normal	p.Thr404fsX30	-	
	II:3	Normal	-	-	
	III:1*	Severe PCG	p.Thr404fsX30	162-kb del(2p21.1)	
ICO-64	I:1	POAG	-	-	*Mild phenotype under medical treatment
	I:2*	POAG	p.Tyr81Asn	-	
	II:2	Normal	-	-	
ICO-65	I:1	ARS	p.Ala443Gly	-	
	II:1	ARS	-	-	
ICO-84	III:1	Normal	p.Glu229Lys	-	*Index patient with extremely severe phenotype in both eyes. One eye LP after repeated trabeculectomies and cyclodestruction and the other eye HM after trabeculectomy and tube implantation
	IV:1	Normal	p.Glu229Lys	-	
	IV:3	Mild JOAG	p.Glu229Lys	p.Arg368His	
	IV:4*	Severe PCG	-	p.Arg368His	
	IV:5	Severe PCG	p.Asp449fsX6	p.Arg368His	
	V:1	Normal	-	-	
	V:2	Normal	-	-	
	V:3	Normal	-	-	
	V:4	Normal	-	p.Arg368His	
	V:5	Normal	-	p.Arg368His	
	V:6	Normal	p.Glu229Lys	-	
	V:7	Normal	-	p.Arg368His	
ICO-96	G-166	ARS	p.Glu229Lys	-	

Index patients are indicated by an asterisk. VA visual acuity; LP light perception; HM hand movements; RE right eye; LE left eye.

**Table 5:** *CYP1B1* mutations detected in the PCG and ARS families studied from EMEIGG.

Family No.	Patient No.	Phenotype	Mutation 1	Mutation 2	Treatment and prognosis
EMEIGG-10004	IV:1*	PCG	p.Trp57Stop	p.Ser464PhefsX12	One eye badly impaired treated with trabeculectomy and tube implantation, the other with moderate phenotype and good response to goniotomy and trabeculectomy
EMEIGG-12005	I:1	Normal	-	p.Leu277Stop	*Severe phenotype, one eye enucleated after repeated filtering surgeries and the other with advanced damage but IOP controlled medically
	I:2	Normal	p.Gly61Glu	-	
	II:2*	PCG	p.Gly61Glu	p.Leu277Stop	
	II:3	Normal	-	p.Leu277Stop	
	III:1	Normal	-	p.Leu277Stop	
	III:2	Normal	-	p.Leu277Stop	
EMEIGG-12008	I:2	POAG	p.Ala179ArgfsX16	p.Arg368His	I:2, mild POAG phenotype II:1, one eye with severe phenotype (2 trabeculectomies) and the other moderate (2 trabeculectomies) II:2, OHT with initial damage at OCT with normal visual fields, medical treatment initiated
	II:1*	JOAG	p.Ala179ArgfsX16	p.Arg468_Ser476dup	
	II:2	Normal	p.Arg368His	p.Arg468_Ser476dup	
	II:3	Normal	-	p.Arg368His	
	III:1	Normal	-	p.Arg468_Ser476dup	
	III:2	Normal	-	p.Arg368His	
	III:3	Normal	-	p.Arg468_Ser476dup	
	III:4	Normal	-	p.Arg468_Ser476dup	
EMEIGG-20004	I:1	Normal	-	-	*Unilateral PCG with severe damage treated with trabeculectomy and tube implantation
	I:2	Normal	-	p.Arg368His	
	II:1	Normal	-	p.Arg368His	
	II:2*	PCG	-	p.Arg368His	
HCL-21	G-271*	POAG	p.Tyr81Asn	-	Severe bilateral damage treated with tube implantation but presence of neovascular glaucoma secondary to diabetic retinopathy

Index patients are indicated by an asterisk.

### Phenotype-genotype correlation in myocilin carriers

The p.Gln368Stop mutation is a null allele previously reported in other glaucoma survey populations, and which caused POAG in families ICO-4 (Figure 1) and EMEIGG-23001 (not shown). In family ICO-4, the phenotype observed was variable. Thus, index patient II:3 presented with glaucoma with a severe phenotype that required aggressive surgical treatment but still had a bad visual prognosis. However, cosegregation of p.Gln368Stop in the family detected a carrier of the mutation (III:1) who remains unaffected, while member III:2, clinically observed to have OHT and thick pachymetry readings, did not carry the mutation; glaucoma treatment was therefore withdrawn in this patient. The index patient from family EMEIGG-23001 presented a mild phenotype of POAG requiring just one hypotensive agent. However, as he was recently diagnosed during his third decade of life, the severity, rate of progression of his glaucoma, and need for additional drugs or surgery remain to be determined. Other family members were not available for study.

Two mutations in consecutive residues of *MYOC*, p.Val426Phe (family ICO-45) and p.Ala427Thr (ICO-24), previously reported as causing glaucoma, were detected in two index patients (Figure 1). Patient IV:1 of family ICO-45 with p.Val426Phe showed a JOAG phenotype with an aggressive type of glaucoma that required surgical treatment at an early age of life. Unfortunately, although there are many affected patients in this family, no other members were available for the study. Patient II:1 in family ICO-24 (Figure 1), who carried the mutation p.Ala427Thr, showed normal tension glaucoma with a poor prognosis.

The mutation p.Thr293Lys was present in patient II:3 of family EMEIGG-8008, with a mild form of POAG. Unfortunately, no relatives wanted to participate in this study, despite the high incidence of glaucoma in this family. This mutation was described for the first time [22] in a patient with normal tension glaucoma.

The previously reported p.Tyr479His mutation [24] was found in patient I:1 of family EMEIGG-12014 (Figure 1), who presented POAG at 32 years of age but responded satisfactorily to antiglaucoma drugs. The proband presented with initial glaucomatous damage in one eye, requiring filtrating surgery, and ocular hypertension in the fellow eye. We analyzed the patient’s son (II:1) and daughter (II:2), and both are carriers of the mutation. They remain unaffected, but follow-up visits are mandatory in these patients to detect the onset of the disease, especially during the third decade of age.

A p.Glu352Lys variation was found in the index patient of family EMEIGG-21007 (not shown), with a severe form of PCG. This mutation has been previously described in a single case in a Canadian series of patients, but the pathogenicity remains unclear [20].

### Phenotype-genotype correlation in cytochrome P450, family 1, subfamily B, polypeptide 1 carriers

In family ICO-3 (Figure 2), we found the homozygous deletion c.1064_1076del that creates the frameshift mutation p.Arg355fsX69, previously reported [26], in monozygotic twins (II:1, II:2) with severe PCG who required several surgical interventions and cyclodestruction in one eye; the other eye presented a milder condition and was well controlled with goniotomies and medication. Both parents were asymptomatic and heterozygous carriers of the mutation. To discard the possibility that the homozygous mutation in the monozygotic twins was due to the loss of a paternal allele, quantification of the copy number of *CYP1B1* was performed using multiplex ligation-dependent probe amplification analysis. The result showed two *CYP1B1* copies in both twins. Additionally, member I:2 carried the mutation c. 685G>A, which creates the change p.Glu229Lys in compound heterozygosis with p.Arg355fsX69. However, this individual remains unaffected. Previously, the p.Glu229Lys variation has been reported as a hypomorphic allele [35].

The index patient II:1 of family ICO-12 (Figure 2) was compound heterozygous for the previously reported duplication c.1198_1207dup, which generates the frameshift mutation p.Thr404fsX30 [27], and the known missense mutation c.1405C>T (p.Arg469Trp) [36]. This patient had severe PCG that needed Molteno tube implantation in both eyes during childhood. The proband’s parents (I:1, I:2) and brother (II:2) are carriers of one of the two mutations and remain asymptomatic.

**Figure 3:**
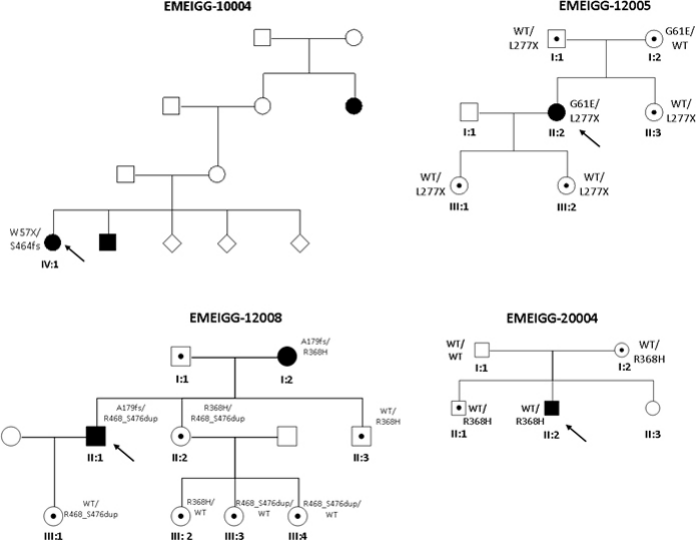
Pedigrees of glaucoma families from the Estudio Multicéntrico Español de Investigación Genética del Glaucoma (EMEIGG) carrying mutations in *CYP1B1*. Filled symbols represent affected subjects, and unfilled symbols unaffected subjects. Squares indicate men, and circles women. Arrows represent the index patients. WT depicts the wild-type allele.

The mutation p.Gly61Glu was detected in compound heterozygosis [36] with p.Leu277Stop in patient II:2 of family EMEIGG-12005 (Figure 3) who showed a severe phenotype. Individuals of this family carrying one of these two mutations were unaffected.

In patient II:1 of family ICO-30 (Figure 2), we detected the mutations p.Arg355fsX69 and p.Thr404fsX30 in compound heterozygosis. This patient had severe PCG in both eyes requiring bilateral tube implantation. The parents of the patient, who carry one of these mutations, are unaffected. Members II:2 and II:3 were not available for this study.

In family ICO-47, patient III:1 (Figure 2) presented the p.Thr404fsX30 mutation in homozygosis. He had severe PCG that required goniotony and tube implantation bilaterally. Analysis of his relatives revealed that his father (II:1) did not carry a mutation and that his mother (II:2) was a heterozygous carrier of mutation p.Thr404fsX30. After discarding false paternity and maternal disomy, we studied this case more deeply to analyze a possible deleted allele in patient III:1. Using gene dosage assessment and analysis of 23 markers of chromosome 2 and four *CYP1B1* intragenic polymorphisms in all members of family ICO-47, we found that the index patient (III:1), his father (II:1), and his grandfather (I:1) harbored a 162 kb-deletion in region 2p21.1 in heterozygosis. Using long-range PCR and primer-walking (Appendix 1), we characterized the deletion, which was between positions 38,187,289-38349505 of genomic DNA from chromosome 2 (ref. NC_000002.11); between these positions, a sequence of 70 bp was inserted. This 70-bp fragment has 98.5% identity with positions 37,824,812-37824872 of chromosome 2. The 162-kb deletion comprises a partial portion of the *FAM82A1* gene (all except 10 kb of the 5′ region of the gene), the complete LOC100288457 gene, and the complete *CYP1B1* gene. The father and grandfather remain asymptomatic.

The index patient of family ICO-39 (not shown) had JOAG and carried the known mutations c.1103G>A (p.Arg368His) [37] and p.Thr404fsX30. His relatives were not available for this study. In index patient IV:4 of family ICO-84 (Figure 2), with severe PCG, we detected only the known mutation p.Arg368His in heterozygosis. When we performed the analysis in her relatives, we detected the previously reported missense mutation p.Glu229Lys in patient IV:3, who had a mild form of JOAG, in compound heterozygosis with mutation p.Arg368His. We found that p.Glu229Lys and p.Arg368His were also present in heterozygosis in other asymptomatic relatives. p.Glu229Lys has been described in POAG and juvenile-onset glaucoma, as a hypomorphic allele and reduces the abundance of the enzyme [13]. In patient IV:5, we found the mutation p.Arg368His with the previously reported mutation c.1344_1345delG (p.Asp449fsX6) [27], in compound heterozygosis. This patient had severe PCG. The Arg368His mutation was also found in heterozygosis in patient II:2 of family EMEIGG-20004 (Figure 3). The p.Arg368His mutation has been associated with *GLC3A* and glaucoma, and could act in digenic early-onset; this mutation may act as a modifier of the *MYOC* mutant phenotype [13]. However, no *MYOC* mutation was found in carriers of the Arg368His mutation.

The previously reported mutation c.171G>A (p.Trp57Stop) [28] and the novel variation c.1385_1390insT (p.Ser464PhefsX12) were found in patient IV:1 of family EMEIGG-10004, who has PCG. p.Trp57Stop has been previously described [38] in a patient with Peters’ anomaly, a congenital abnormality that shows a wide range of histopathological changes, including glaucoma (more than 50% of subjects develop glaucoma in childhood). In this family, no other relatives were available for the study.

In family EMEIGG-12008, we found that the index patient (II:1), who has JOAG, was a carrier of the mutation c.534_535delG (p.Ala179ArgfsX16) and the novel duplication c.1403_1429dup that creates the variation p.Arg468_Ser476dup. An additional mutation p.Arg368His together with p.Ala179ArgfsX16 was found in his mother (I:2), who has POAG. The combination of these two mutations may explain the phenotype differences in the mother and the son. Curiously, patient II:2 is a carrier of mutations p.Arg368His and p.Arg468_Ser476dup; at the moment, she remains asymptomatic, but she is being examined more frequently as some initial peripapillary nervous fiber loss has been documented with OCT. Presumably, she could develop glaucoma in the future.

Finally, the change c.241T>A (p.Tyr81Asn) was found in three index patients with POAG: patient III:2 of family ICO-18 (Figure 2), patient I:2 of family ICO-64, and the index patient of family HCL-21 (not shown). However, in these families no second mutation in *CYP1B1* was found. The p.Tyr81Asn mutation was first described in a family with POAG [30] and seems to be pathogenic. However, in family ICO-18, although the individual III:5 had an aggressive form of pseudoexfoliative glaucoma that required trabeculectomies in both eyes, one carrier of the mutation (III:2) has POAG, and another, IV-4, has pigmentary dispersion syndrome (PDS) and OHT. Patient IV:1, who does not carry the mutation, has OHT. The p.Tyr81Asn variation proved to be uncertain as the cause of glaucoma in these families.

### Analysis of cytochrome P450, family 1, subfamily B, polypeptide 1 mutations in patients with Axenfeld-Rieger syndrome

Two mutations were detected in two index patients with ARS. Patient I:1 (family ICO-65) presented the previously reported PCG-associated mutation c.1328C>G that generates the change p.Ala443Gly [39], but his son, II:1, does not carry the variation. Both had ARS. This demonstrates that in this family the ARS phenotype is probably not associated with this variation. p.Ala443Gly has been described in GLC3A and POAG, but the variation’s pathogenicity has not been demonstrated [12]. We also detected the variation p.Glu229Lys in the index patient of family ICO-96 (not shown), who had ARS. Unfortunately, no other relatives were available for genetic screening.

## Discussion

A genetic base for glaucoma pathogenesis has been established [1,8,40]. In this study, we included Spanish index patients with a positive family history of glaucoma or OHT. Whenever possible, affected relatives of index cases were included. All the patients and asymptomatic relatives included in this study underwent a complete ophthalmologic examination. This enabled us to classify the glaucoma patients in the different phenotypes shown in Table 2. To investigate the incidence of mutations in genes associated with the different phenotypes of glaucoma, we checked for mutations in *MYOC* and *CYP1B1* in a large cohort of a Spanish population.

Mutations in *MYOC* have been reported to cause POAG through a dominant trait. *MYOC* is the major candidate gene for glaucoma thus far [41]. However, in our survey, we detected relatively few mutations in this gene (4.4% in the families studied) causing familial glaucoma.

We found a novel *MYOC* variant, p.Glu218Lys, in a patient with POAG with a mild glaucoma phenotype that was also detected in her daughter, who had OHT with initial OCT alterations as the only symptom of glaucoma to date. This variation has been previously described in monkeys [42], in an experiment analyzing the *MYOC* gene in monkey and human steroid-induced ocular hypertension. Steroid-response (increased IOP) was assayed with the administration of dexamethasone in cynomolgus monkeys. In this study, when variations found in monkeys were analyzed individually or as a group, there was no significant association with the steroid response. The variation p.Glu218Lys was found in heterozygosis in a nonresponder monkey. The monkey and human genes predict proteins that are 97% identical, but as observed in human studies, where most of the responders who are analyzed carry mutations in *MYOC*, mutations in this gene do not appear to be a common cause of the steroid response in monkeys. This mutation has not been previously described in humans, but in monkeys, it is not associated with an increase in IOP in response to steroids (a situation that can be compared with glaucoma). Moreover, the SIFT algorithm predicted this variant was benign, but the PolyPhen prediction was possibly damaging. Therefore, the p.Glu218Lys variant is an uncertain cause of POAG. The second novel variation detected in our survey, p.Lys39Arg, was present in the index patient and his unaffected relatives and predicted by SIFT and PolyPhen as tolerant. Therefore, it seems unlikely to be the cause of glaucoma in this family.

We observed significant differences in the phenotype expression of glaucoma caused by the mutation p.Gln368Stop in *MYOC.* This mutation causes a premature terminal codon (PTC), and although located in the last exon of the *MYOC* gene, whether there is differential expression of the mutant allele in carriers, due for example to a nonsense-mediated messenger RNA decay (NMD) mechanism, remains to be determined. This is the most frequent *MYOC* mutation reported in Caucasian patients and normally causes mild phenotypes, unlike what was seen in patient II:3 (ICO-4), who had a severe form of glaucoma. The other carrier of this mutation in family EMEIGG-23001 (not shown) was too young to determine the type and rate of progression of his otherwise mild phenotype. The family ICO-4 (Figure 1) is a good example of how genetic evaluation can be considered a tool for diagnosing glaucoma, just like the visual field or OCT examinations. Patient III:2 was being carefully monitored, undergoing frequent glaucoma tests, and was receiving prophylactic hypotensive treatment even though all the tests were unremarkable, due to the aggressive form of glaucoma present in her relative II:3. The patient felt less anxious if she was treated and evaluated frequently. The absence of a mutation in this patient allowed us to withdraw the treatment and reduce the frequency of the visits, resulting in considerably less anxiety for the patient.

We detected a high percentage of mutations in *CYP1B1*, which causes glaucoma in families with PCG but not in other glaucoma phenotypes. This suggests that *CYP1B1* is the main known candidate gene causing PCG in our population, with a low or null contribution in other glaucoma phenotypes. In only one PCG family did we detect the mutation in *CYP1B1* in homozygosis (p.Arg355fsX69), in monozygotic twin girls. Their mother is a double heterozygote for this mutation and p.Glu229Lys. We also found this mutation together with the recurrent (see below) p.Arg368His in a patient (IV:3 of family ICO-84) with JOAG and in a patient with ARS. Moreover, p.Glu229Lys has been previously reported as a hypomorphic variant. Interestingly, the double heterozygous (p.Glu229Lys and p.Arg355fsX69) mother of the two twin girls remains unaffected. This suggests a late-onset glaucoma effect exerted by these mutations or a case of incomplete penetrance.

The mutation p.Thr404fsX30 was detected in homozygosis in a patient with PCG. However, analysis of the parents of this patient showed the mutation only in his mother. Analysis of the *CYP1B1* alleles in the patient’s father showed a deletion of 162 kb in region 2p21.1 in heterozygosis. This deletion was also present in the patient’s grandfather. Characterization of the deletion showed the partial elimination of the family with sequence similarity 82, member A1 (*FAM82A1*) gene and total deletion of the LOC100288457 and *CYP1B1* genes in heterozygosis in both individuals (Appendix 1), but there were no clinical symptoms.

We found the recurrent p.Arg368His mutation of *CYP1B1* in four independent families with glaucoma. However, we also found phenotype differences in members of the same family carrying this mutation. Thus, in family ICO-84, patient IV:3, a double heterozygote for p.Arg368His and the missense mutation p.Glu229Lys, shows a mild JOAG phenotype, while patient IV:5, with a severe PCG phenotype, carries the nonsense mutation p.Asp449fsX6 in compound heterozygous with the mutation p.Arg368His, suggesting a deeper effect for the nonsense mutation. We also detected the p.Arg368His mutation in patient II:2 of family EMEIGG-20004, who has a severe PCG phenotype, but no other variation was found in the coding or flanking sequences of *CYP1B1* analyzed. Furthermore, in family EMEEIG-12008 we found a patient with POAG (I:2) with a double mutation in *CYP1B1*, p.Ala179Argfsx16/p.Arg368His, while member II:2, who carries the compound heterozygous mutations p.Arg368His/p.Arg468-Ser476dup, remains asymptomatic. This previously unreported variant creates a duplication of seven amino acids, where the heme binding site lies at position 470. However, the pathogenesis proved uncertain. Our results for p.Arg368His, and in general for double heterozygotes for *CYP1B1* mutations, suggest a more severe phenotype for glaucoma when a null allele due to a nonsense mutation is present.

We observed differences in phenotypes between individuals presenting only one mutation in *CYP1B1*. A possible cause for PCG, other than double mutant alleles of *CYP1B1,* is plausible, and whether a mutation in *CYP1B1* contributes to the glaucoma phenotype [42] in these patients remains to be investigated. In fact, we found one case (EMEIGG-21007, not shown) with a severe form of PCG with the mutation p.Glu352Lys in *MYOC* although no mutation in *CYP1B1* was found. A possible polygenic mechanism has been suggested to explain the variable phenotype in glaucoma patients [43,44], even between members of the same family. Heterozygous changes in *CYP1B1* have been suggested as a possible modulator of glaucoma phenotype [43-45] in association with mutations in other genes like *MYOC*. However, in our survey we did not find a patient with a double mutation in *MYOC* and *CYP1B1*. Although a major genetic cause of glaucoma remains to be discovered, we believe that mutational screening should be implemented in routine glaucoma clinical practice as it can help the clinician decide on the therapeutic regimen, can provide a more accurate visual prognosis, and may also lead to the diagnosis of new family cases and the establishment of genetic counseling.

## Appendix 1. Supplemental methods.

To access the data, click or select the words “Appendix 1.”
